# Reviews

**Published:** 1862-02

**Authors:** 


### REVIEWS.

Baron Larrey.—*A Lecture by* D. Hayes Agnew, M. D., *Surgeon to the Philadelphia Hospital, Lecturer on Anatomy, &c. Published for the Class. Philadelphia:* Lindsay & Blakiston, 1861.

This is a lecture on the life of Baron Larrey, the eminent French Surgeon, delivered by Dr. Agnew to his Class, as an introductory lecture. It details the various incidents in the life, character and professional achievements of the great Military Surgeon. Larrey was appointed Surgeon-Major to the Hospitals of the Rhine in the campaign undertaken by Napoleon the 1st. It was here that he discovered the great defect in the arrangements for affording prompt relief to such as fell upon the field of battle, which led to the invention of the *flying ambulance*, "now so familiar to our own people, and by means of which the wounded are carried from the field during the progress of the fight." During this campaign he invented the lancet-pointed suture needle, with groove leading from the eye, for which was decreed him a gold medal of a hundred livre's value. It was also during this campaign that Larrey was able to convince himself of the value of primary amputations. The lecture describes Larrey's position, duties, suggestions and sanitary regulations not only during the campaign on the Rhine, but also in Corsica, Italy, Egypt, Syria, Boulogne, Ulm, and Austerlitz. Again the campaigns in Saxony and Prussia, Poland and Spain, Austria and Russia, and lastly that in France in 1813. In 1840, when the mortal remains of Napoleon were brought home to France, Larrey participated in the formalities attending that great funeral pageant, which he and his associates designated as their "last campaign."

"Having a wish to visit again the camp, in 1842 he obtained from Marshal Soult, the Minister of War, an order to visit Algeria, and inspect the hospitals of the French there established. Accompanied by his son, they left Paris, and accomplishing the object of his mission, was on his way home, when he was attacked with pneumonia, and expired at Lyons, on the 25th of July, aged 76 years. His remains were taken to Paris, and on the day when they were deposited in the vault, gratuitously prepared by the authorities of Paris, a vast concourse collected to testify their respect for this great man, among whom were the members of the Academy of Sciences, the Society of Medicine, the civil and military authorities, the ancient soldiers of the Empire, and numbers of distinguished citizens. "If ever," said Napoleon, "the military erect a statue, it should be to Baron Larrey, the most virtuous man I have ever known." Posterity is not insensible to the claims of genius, and already two monuments have arisen to the memory of Larrey; one in 1850, in the court of the Val-de-Grace Hospital, and the other in the hall of the Academy of Medicine.

It is very natural when an individual has filled a distinguished place in the world, to desire to know something of his personal appearance and habits of life. Let it suffice for me to say that Larrey was the model of a military surgeon. His person a little obese, was not above the medium height; his head was well set and developed, measuring 590 millimetres, the same as the Emperor's; his face was somewhat oval, and eyes salient, with a countenance expressive of benevolence and energy; which, with a robust constitution and sanguine temperament, admirably qualified him to endure great privation and fatigue. As an operator, he was judicious, but bold and rapid; calm and self-possessed in every emergency; but full of feeling and tenderness. He stands, gentlemen, among military surgeons, where Napoleon stands among generals—*the first, and the greatest.*"

---

*The Placenta, The Organic Nervous System, The Blood, The Oxygen, and the Animal Nervous System, Physiologically Examined. By* John O'Reilly, M. D., *Licentiate and Fellow of the Royal College of Surgeons in Ireland; Resident Fellow of the New York Academy of Medicine; Member of the Medico Chirurgical College of New York; late Medical Officer to the Oldcastle Workhouse and Fever Hospital, England. New York:* S. S. & W. Wood, 389 *Broadway. London:* John Churchill, *New Burlington Street.*

This is truly an original book, and in it many things are explained which have formerly been denied or regarded as inexplicable. The chapter on the Anatomy and Physiology of the Placenta is very interesting and instructive. The vascular and nervous connections, between the mother and child is illustrated and demonstrated, and a reasonable explanation given of the mode by which impressions are communicated to the fœtus from the mother. The anatomy and functions of the Organic Nervous System are fully, ingeniously, and so far as is known, truthfully given. It is shown to act a very important part in the economy of life. This is a book which highly commends itself to the consideration of the anatomist and physiologist. The author shows himself an original thinker and investigator, and we earnestly commend this book to the study of the profession.

